# Arteriovenous access in hemodialysis: A multidisciplinary perspective for future solutions

**DOI:** 10.1177/0391398820922231

**Published:** 2020-05-22

**Authors:** Bernd Stegmayr, Christian Willems, Thomas Groth, Albino Martins, Nuno M Neves, Khosrow Mottaghy, Andrea Remuzzi, Beat Walpoth

**Affiliations:** 1 Department of Public Health and Clinical Medicine, Umeå University, Umeå, Sweden; 2 Department of Biomedical Materials, Institute of Pharmacy, Martin Luther University of Halle-Wittenberg, Halle, Germany; 3 Interdisciplinary Center of Material Research, Martin Luther University of Halle-Wittenberg, Halle, Germany; 4 3B’s Research Group, I3Bs–Research Institute on Biomaterials, Biodegradables and Biomimetics of University of Minho, Headquarters of the European Institute of Excellence on Tissue Engineering and Regenerative Medicine, AvePark–Parque de Ciência e Tecnologia, Barco, Portugal; 5 Department of Physiology, RWTH Aachen University, Aachen, Germany; 6 DIGIP, University of Bergamo, Bergamo, Italy; 7 Department of Cardiovascular Surgery (Emeritus), University of Geneva, Geneva, Switzerland

**Keywords:** Arteriovenous access, artificial kidney, apheresis and detoxification techniques, hemodialysis, dialysis access, arterial grafts, vascular grafts, polymer membranes, biomaterial surface characterization, blood–material interactions, tissue engineering, wall shear stress

## Abstract

In hemodialysis, vascular access is a key issue. The preferred access is an arteriovenous fistula on the non-dominant lower arm. If the natural vessels are insufficient for such access, the insertion of a synthetic vascular graft between artery and vein is an option to construct an arteriovenous shunt for punctures. In emergency situations and especially in elderly with narrow and atherosclerotic vessels, a cuffed double-lumen catheter is placed in a larger vein for chronic use. The latter option constitutes a greater risk for infections while arteriovenous fistula and arteriovenous shunt can fail due to stenosis, thrombosis, or infections. This review will recapitulate the vast and interdisciplinary scenario that characterizes hemodialysis vascular access creation and function, since adequate access management must be based on knowledge of the state of the art and on future perspectives. We also discuss recent developments to improve arteriovenous fistula creation and patency, the blood compatibility of arteriovenous shunt, needs to avoid infections, and potential development of tissue engineering applications in hemodialysis vascular access. The ultimate goal is to spread more knowledge in a critical area of medicine that is importantly affecting medical costs of renal replacement therapies and patients’ quality of life.

## Introduction

The number of patients with end-stage renal disease (ESRD) in need of renal replacement therapy by dialysis and especially hemodialysis (HD) is rising and was 2.5 million patients worldwide in countries having registers in 2015.^1^ In patients who will be offered HD, the European guidelines^2^ recommend that the ideal vascular access (VA) should allow cannulation using two needles. One access, the arterial line, allows blood to enter into the extracorporeal circuit (ECC) including the dialyzer. The other access, the venous line, allows blood within the ECC to return back to the patient. The arterial access (arteriovenous fistula (AVF) or arteriovenous shunt (AVS)) should deliver a minimum blood flow of at least 300 mL/min through the artificial kidney and be resistant to infection and thrombosis and should have minimum adverse events. The first option for the construction of a VA is the creation of an autogenous AVF. The principle of venous preservation dictates that the most distal AVF possible should usually be performed. The secondary option is a prosthetic AVS usually made by synthetic graft material (arteriovenous graft (AVG)). The tertiary option is a central dialysis catheter that is partly placed in a subcutaneous tunnel (tunneled dialysis catheter (TDC)).^3–5^ The reason for creating autogenous AVFs is that observational studies show a lower incidence of post-operative complications and fewer endovascular and surgical revisions for AVF failure in comparison with AVGs. In addition, the use of TDCs results in a significantly higher morbidity and mortality rate. The risk of hospitalization for VA-related reasons and particularly for infection is highest for patients on HD with a catheter at initiation and throughout follow-up.^2^ When HD is the choice, early referral to the nephrologist enables an early plan for venous preservation that is a substantial part of pre-dialysis care and education. Such approach may minimize the use of catheters and reduce catheter-related morbidity and hospitalization.^2^ When using autogenous AVFs, observational studies show a lower incidence of post-operative complications and fewer endovascular and surgical revisions for AVF failure in comparison with AVGs.^2^ When TDC is the choice, a significantly higher morbidity and mortality rate may be expected.

The placement of a central dialysis catheter is mainly used in emergency situations and in chronic HD if VA by AVF or AVS is not plausible.^3–5^ AVF is used more frequently, also in elderly patients, in Japan, while in Europe and United States often HD is initiated by the use of an AVS or TDC.^6^ In addition, over time, more upper-arm AVF and AVG are placed especially in Europe and United States.^6^ This is a caution since geographic areas that have a higher prevalence of the use of AVF report better survival data.^7–9^ Even if there exist only few differences in genetics and baseline renal diagnosis, the prevalence of AVF and AVS versus TDC varies between countries and even between 40% and up to 80% in different centers within the same country, such as Sweden.^10^ Since patient demography can be expected to be similar within the country, this indicates that the reasons for those marked differences depends not only on the conditions of the vessels but also on the preference of the local physician’s prescription of type of access and the skills of the local surgeons to place AVF and AVG.^11,12^

**Figure 1.**
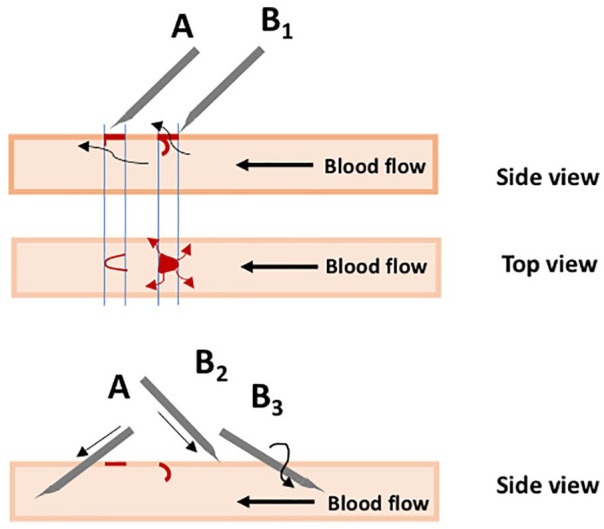
If a cutting needle is used in an AVF or AVS with the edge downward and against the blood flow direction, there will be a flap of the vessel wall that will obstruct the vessel and keep an open area at the site of injection that increases the risk of hematoma. If the edge is turned up-side down, the flap of the vessel will tighten as a lid. Locating the needle with the flat side down during HD minimizes the risk for puncture of the opposite wall.

Puncture techniques may interfere with AV patency. By changing the position of the punctures each time, a “rope ladder” technique is used. A less painful puncture performed in the same holes as before is the “buttonhole technique.” The rope ladder technique is generally recommended for AVS grafts (starting between 4 and 6 weeks after insertion), while for AVF (in mean starting 2 months after surgery) both techniques are mentioned and “buttonhole” is preferred.^4^ While Chan et al.^13^ could not find different outcomes for the techniques for primary patency or episodes of bacteremia, others showed the “buttonhole technique” to imply a substantial risk for access-related infections.^14^ For vascular grafts, such infections can be locally invasive and difficult to cure with a consequence of intermittent seeding of bacteria into the blood with septic reactions.^15,16^ The disadvantage of the puncture technique with a cutting needle in synthetic vascular grafts is shown in Figure 1. Thereby open holes appear when material is cut out of the graft (see later).

Following a multidisciplinary approach, we reviewed the conventional solutions for AVF and AVS clinical management, as well as future perspectives. The aim is to recapitulate the vast and interdisciplinary scenario that characterizes HD VA creation and function, since adequate access management must be based on knowledge of the state of the art and on future perspectives. We also discuss recent developments to improve AVF creation and patency, the blood compatibility of AVS, need to avoid infections, and potential development of tissue engineering applications in HD VA. The ultimate goal is to spread more knowledge in a critical area of medicine that is importantly affecting medical costs of renal replacement therapies and patients’ quality of life.

## Location of AV access

**Figure 2.**
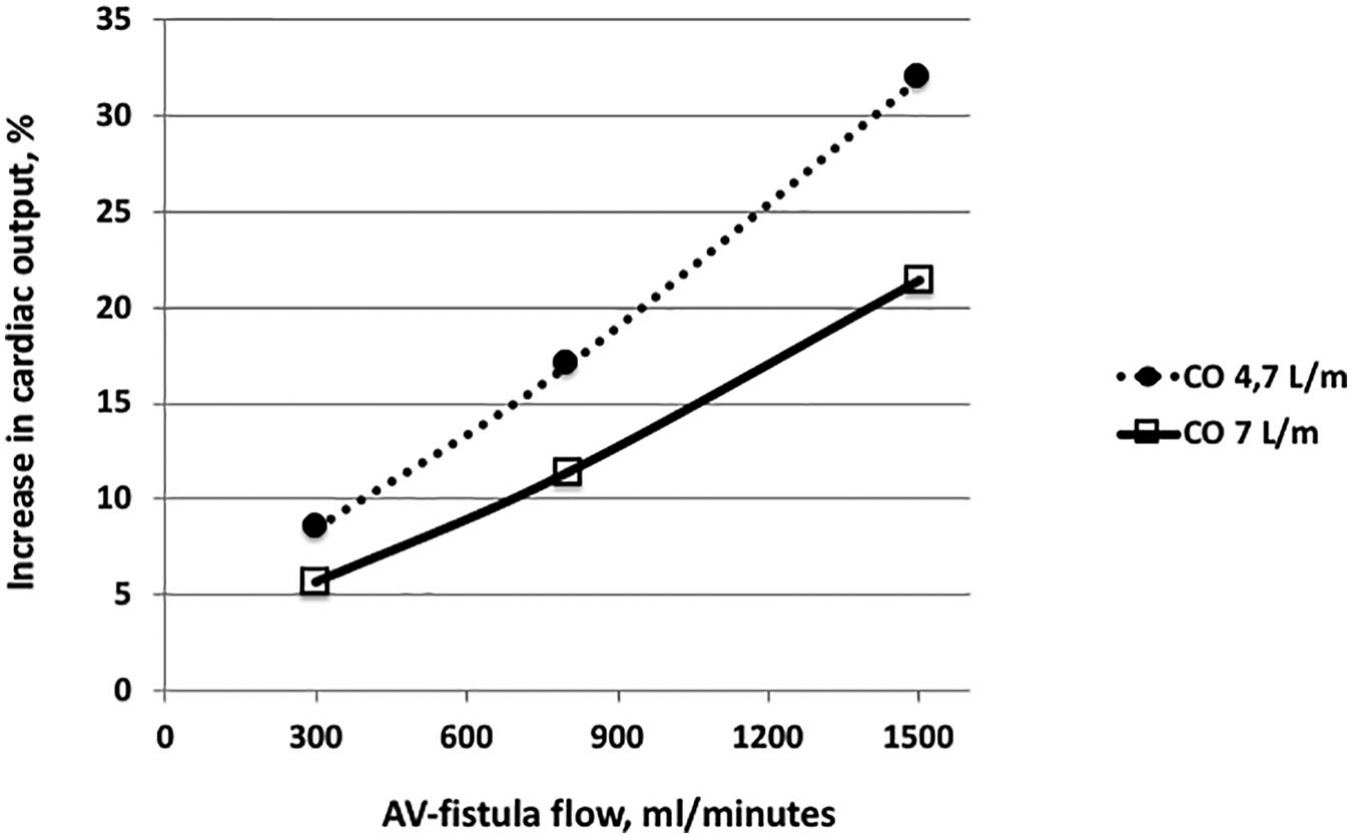
Change in cardiac output in relation to AVF blood flow in patients of either 50 kg BW and 161 cm height (open square, hatched line) or 90 kg BW and 185 cm (open triangle and filled line). Calculations are based on Jegier et al.^21^

The preferred location of the AV access is an autogenous distal wrist radiocephalic access performed at the non-dominant arm. If this is not possible, more proximal options are selected such as mid-forearm to the elbow area before placement in the upper-arm region (brachial-cubital/cephalic/basilic AVF).^4,8^ During recent years, a larger proportion of AVFs and AVGs are placed in the upper arm, particularly in patients within Europe and United States.^6^ Upper-arm placements result in high return rate of shunted blood volumes per minute to the heart. To avoid secondary cardiac strain,^17^ or even congestive heart failure,^18^ the shunted blood volumes have to be proportional to the body size and the condition of the heart of the patient (Figure 2). Another consequence of the upper-arm AVF or AVS is that it causes a more extensive surgical approach for exploration of the vessels. This may be weighed against the alternative of TDC where the risk of subsequent infections and flow problems is significantly increased.^8,19,20^ Placement of AVF is made either as side-to-side of artery and vein or end-to-side of the vein/graft to the artery.^4,8^ AVSs are normally placed end-to-side to the artery and end-to-side or end-to-end to the vein. Vessel diameter and condition are important for outcome.

## Risk factors for AV access dysfunction

The incidence of non-maturation of an AVF varies between 20% and 60%,^12,22–24^ mostly leading to further surgical or catheter interventions in order to attain a VA that enables HD. The main difficulties can be caused by either too low flow or clotting that develops predominantly upon stenosis due to neointimal proliferation.^23^ Vascular dysfunction may arise within the feeding artery and is considered to be related to factors such as age, diabetes mellitus, hypertensive diseases, uremia, tobacco use, and inflammation.^12,23,25^ Anastomosis-related problems are considered mainly to be due to surgical measures and once established to wall shear stress and turbulence.^12^ Some reports attribute those problems to the angle of the fistula toward the artery,^12^ but this hypothesis was not confirmed by others.^25^ Post-anastomosis-related problems are stenosis and thrombosis such as in elderly patients (⩾65 years), those with coagulation abnormalities, hypotension, and smoking^12,26,27^ besides clamping in conjunction with compression, repeated needle punctures, and local hematoma.^12,23,28,29^ All these factors upregulate inflammatory cytokines that are associated with matrix deposition and increased risk of thrombosis. While patients with diabetic kidney disease have worse vascular conditions,^25,30^ those with polycystic kidney disease tend to get larger diameter sizes of the AVF.^31^ All these problems may lead to radiological investigations and other interventions such as percutaneous balloon dilation, endovascular stenting, thrombolysis, or reconstructive surgery.^25^ Can such problems be expected and prevented?

## Preoperative measures

To clarify conditions before surgery, besides clinical judgments, it is recommended to perform vascular mapping by ultrasound, and eventually fistulography, angiography, or computer tomography with angiography.^32^ Patient age, cardiovascular condition, and clinical history should all be considered before the intervention. The non-dominant arm should be preferably used as access. Blood samples should be taken, when possible, from the dorsal vein of the other hand. A protection of the vascular system includes avoidance of placement of subclavian central catheters that constitute a high risk of upper-limb proximal vein stenosis.

## AVFs

The high incidence of non-maturation of an AVF indicates that the surgery should only be performed by practitioners with more than 25 AVFs created during training.^11^ This operation is normally performed with microscope or magnifying loops since the thinnest sutures (less than 6-0) are used in order to prevent later complications such as early thrombosis or late intimal hyperplasia.^33,34^ Vessels are only handled by the adventitia, and the forceps must never grasp the intima. High-pressure clamps must be avoided.^35^ Loops of sutures that retain in the blood stream should be minimized to avoid turbulence, fibrosis, and clots.

Another approach may be to create percutaneous arteriovenous fistula (pAVF) such as using the Ellipsys(R) VA system.^36^

Furthermore, the calibration of the fistula requires experience in order to avoid a low-flow situation which may result in a non-maturation of the fistula or a high-flow situation which may result in cardiac overload, eventually leading to heart failure (Figure 2).

**Figure 3.**
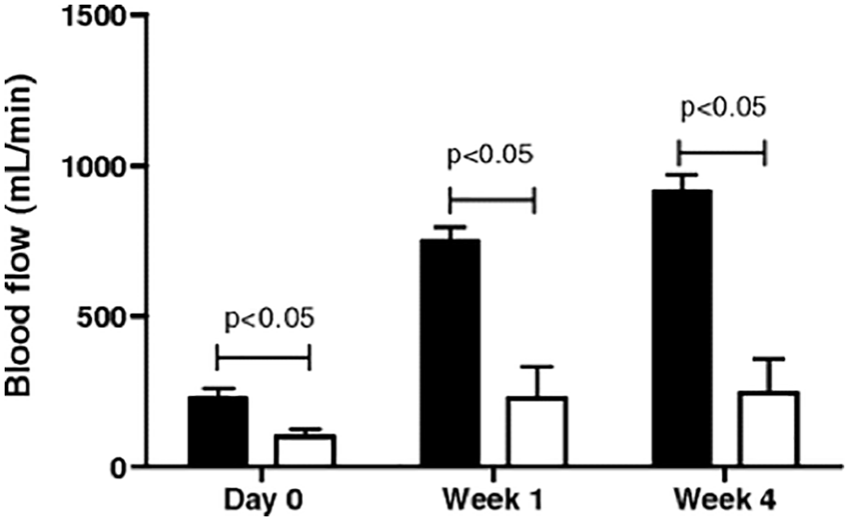
A flow greater than 120 mL/min at the time of surgery results in better maturation rate of the fistula as shown by Saucy et al.^37^ Blood flow (in mL/min) in functioning (black box) and non-functioning radiocephalic AVF (white box).

To prevent primary non-functioning AVF, several investigators assessed the blood flow intra-operatively of the completed fistula with transit-time flow measurements (TTFM). This technique enables to measure an instant flow in an artery or vein and therefore to correct the fistula if it has a too high or too low flow. A flow greater than 120 mL/min at the time of surgery has been shown to have a better maturation rate of the fistula when compared to lower flow values, as shown in Figure 3.^37^ In the case of too high AVF flow, with risk of cardiac overload, the TTFM is used intra-operatively to adjust the flow to about 400 mL/min for autologous fistulas and to about 600 mL/min for prosthetic shunts.^38^

Once the AVF is functioning post-operatively, the patency rate of AVF varies with primary 1-year patency in the order of 60%–70%.^22–24^ The late failure of AVF is mainly related to intimal hyperplasia at the anastomosis between artery and vein and/or thrombosis in low-flow areas in the venous side of the anastomosis. Failure may also be initiated by repeated puncture of the arterialized vein segment. However, secondary patency, even after several years, may be kept in this level, using repeated radiological interventions^25,39^ that seem to be more successful with drug-eluting balloons.^39^ AVF and AVS monitoring using ultrasound more extensively prevent complete access closure and allow timely planning of radiological intervention or surgical revision. Less used to detect VA dysfunction is the monitoring of venous pressure during the dialysis session.

Investigations comprising the use of an external stent can help maintain an optimal anastomosis angle after AVF surgery and during vessel remodeling.^40^ The benefit of an AVS is that the size and flow of the fistula can be estimated by the choice of the surgeon. This may prevent too large AV-flow and prevent subsequent congestive heart failure. Stents made of a fine nitinol mesh^2^ have been used for access stenoses with mixed results.^41–43^

## Measures to maintain VA functions

If the diameter of the vessels is too narrow, it may be increased by physical training of hand and arm muscles. The patient can increase its lower-arm blood flow by repeatedly manually compressing an elastic ball.^4^ Besides the possible protective benefits by angiotensin-converting enzyme (ACE)-inhibitors, heparin, and antiplatelet drugs,^12^ the illumination using far infrared light supports vascular flow^44–46^ and helps to increase the vascular diameter.^44,45^ Despite all such measures as well as repeated interventions, the venous system may get insufficient as an AVF. Since AVF is not possible to be placed in numerous patients, therefore AVS is the alternative.

## AVSs

When AVF is no further option, an AVS can be considered. Again, starting from distal connecting to the radial artery end-to-side in the forearm; the next option is more proximal in the cubital area and eventually toward the upper arm. The shunt consists of a vascular graft that is used either as a straight segment or as a loop in the subcutaneous position. In clinical practice, synthetic grafts are dominant compared to tissue-engineered and biological grafts. Using synthetic AVS grafts is a less viable option than AVF. Even when using optimal surgical insertion technique, patency problems appear with the synthetic material, which raises the interest in tissue-engineered vascular grafts (TEVG). The primary patency rate for AVS at 1 year is around 50%^47^ and, in some studies, the failure rate increases to 0.8–1.0 events per patient per year.^24^ Many HD patients require an exchange of the AVS after 12 months.^48^ After 2 years, half of all the AVS are unfortunately non-functioning and alternate access solutions have to be considered. Why is the survival of the AVS so limited? Below we will discuss the pathophysiology of AVS application and the materials currently used in the clinical practice.

## Pathophysiological considerations of blood–biomaterials interactions

The development of stenoses and thromboses can be seen as a result of a triad of interaction between (1) biomaterial used; (2) flow and blood properties such as shear rate and stress, flow rates oscillations, and backflow besides the interference of the uremic condition, coagulation, and inflammation; and (3) the geometrical shape of vessels and grafts regarding, that is, outer and inner diameter, length, and curvature in relation to anticoagulation conditions.^49^

The synthetic grafts used for access have similar physicochemical properties as the synthetic dialysis membrane. Hence, both materials used for HD and AVS have to be discussed together, toward minimizing their effects on blood and tissues. Blood–biomaterials interactions are especially studied in settings such as HD^50,51^ and heart-lung-machines.^52^ One should distinguish particularly the hemodynamic differences present in the various artificial organs disciplines when we compare the reported results. Thereby, pump flow rates for extra corporeal oxygenation (ECMO) are more than 3 L/min, while for HD 200–400 mL/min. Other factors such as cannulation technique, time of therapy duration, and its frequency will have different effects on stimulation of platelets, blood coagulation factors, and therefore anticoagulation strategies during the extracorporeal circulation that may also interfere with the VA.

Protein adsorption takes place at the artificial membrane surface. This is a complex process that is affected by factors such as the blood composition and the surface characteristics of the membrane.^53^ The blood–membrane contact causes activation of leukocytes and platelets that support microvascular inflammation and oxidative stress.^54,55^

As far as blood properties, the underlying diseases, uremic toxic substances, and repeated dialyses lead to an acute inflammation added on to a chronic inflammatory condition.^12^ This leads to the activation of immune cells and activation of the coagulation and complement system. These factors contribute to the morbidity of the patients.^53,56–59^ Previous HD membranes like cuprophane were strong inducers of inflammation.^60^ Even the modern membranes, that is, modified cellulose, polysulfone (PS), poly(methyl methacrylate) (PMMA), polyamide (PA), polyacrylonitrile (PAN), polyethersulfone,^61^ or poly(ethylene-*co*-vinyl alcohol) (PEVA)^62^ elicit complement activity and related inflammation.^63^ In contrast to the synthetic graft material of AVS, the dialyzer membrane is normally exchanged after each procedure, enabling the hydrophobic nature of most modern membranes to partly bind complement and other plasma proteins.^64^ However, repeated blood–membrane interaction by the dialyzer during HD also initiates thromboembolism. The activated blood is in a high concentration when it returns from the ECC into the VA where it can promote inflammatory reactions and thromboembolic events. Both the flow rate and the flow regime (laminar versus turbulent) influence platelet activation and release of platelet factor that may lead to thromboembolism.^65,66^ It should be reminded that also the access needles/catheters are “trouble makers,” since they can work as an amplifier, catalyser, or simply trapping of stimulated platelets or other coagulatory reactivations.^65,66^

Although the factors that induce vascular changes, stenosis, and ultimately thrombosis are not fully revealed, it is generally accepted that the development of intimal hyperplasia is the cause of vessel stenosis. Such vascular changes take place especially in the juxta-anastomotic region of the venous outflow track. In these regions, the sudden change in flow direction for high blood flow rate induces two changes from the physiological condition of blood flowing in arterial and venous vessels. The first is that in the external portion of the vein the flow velocity is accelerated, while in the opposite wall flow velocity is reduced and it may oscillate, inducing low and oscillating wall shear stresses. This condition is known to induce endothelial cell (EC) dysfunction, reduction of nitric oxide (NO) production, and several signals within EC that induce proliferation of smooth muscle cells, production of cytokines, and mediators of inflammation. The second type of hemodynamic change that develops after VA creation is the flow instability induced by the massive increase in blood vessel diameter and wall thickness of vein segment that is characterized by fast fluctuations of shear stress acting on the EC. This abnormal condition is also suggested to induce EC dysfunction and potential signal to the underlining smooth muscle cells to remodel the extracellular matrix (ECM) and to proliferate.^23,34,67^

## Types of synthetic AVS

Currently used vascular grafts for AVS are made of non-degradable synthetic polymers such as expanded polytetrafluoroethylene (ePTFE) or polyethylene terephthalate (Dacron), having normally an internal diameter of 5–6 mm. The main advantage of those devices is that they are readily available off the shelf. But, in addition to the previous mentioned difficulties, there are other disadvantages of those devices, for example, size and compliance mis-match. The normal flow rates obtained in those shunts are in the order of 5–600 mL/min.

**Figure 4.**
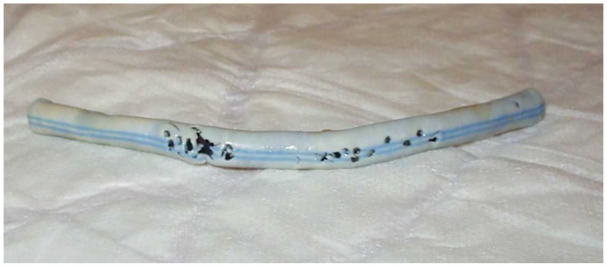
Removed graft visualizes holes after repeated punctures caused by HD access.

Compared to other synthetic polymers, for decades, ePTFE was the material of choice for an AVS, due to its good patency, biocompatibility, and long-term stability. In addition, it is a low-cost and thermally stable material that permits steam-sterilization which facilitates its clinical application. However, a side effect of such synthetic materials is its damage by repeated punctures (Figure 4). It is plausible to assume that the material that is cut-out of the graft by the needle punctures will be deposited in the lungs (an open “foramen ovale” also enables its distribution into the arterial circulation). There it will facilitate local embolies and subsequent infections and scarring. This favors synthetic or biogenic materials that will be absorbed over time.

Indeed, the most pressing issue is graft failure due to thrombosis, which is mainly due to neointimal hyperplasia at the venous anastomosis. The foreign body causes a response of leukocytes, which promotes the growth of smooth muscle cells at the anastomosis between the graft and the blood vessel. The so-caused stenosis leads to higher and irregular shear rates at the affected site, which in turn causes deposition and activation of blood platelets.^68–70^ Anti-platelet drugs are frequently used but they have not been confirmed in its efficacy to prevent AV thrombosis.^71,72^ Other options, therefore, have to be explored. The mentioned activation of coagulation and the onset of the inflammatory processes due to the contact of blood with HD materials may also make a substantial contribution to this process, which has to be considered as well.

From fluid dynamic point of view, the geometrical shape and measures within the graft and ECC are the targets to reduce stagnation points, vortices, and high shear stresses.

Studies have shown that bacterial infection is another serious issue with synthetic grafts.^73–75^ The high risk of infection is presumably due to the porous structure of the graft, which causes a bacterial accumulation while hindering the leukocytes from fighting the infection. Moreover, the quite hydrophobic polymers used for most of the AVSs, such as ePTFE and Dacron, promote adhesion of bacteria and subsequent biofilm formation. This problem is also shared by other polymers of low surface energy.^76^ A direct comparison with bioengineered human acellular vessels shows a much lower risk of infection than for PTFE vascular grafts.^77^ Furthermore, the replacement procedure itself carries many risks, like the possibility of bleeding complications or infection. What options can be used to reduce the risk for these complications?

## Efforts to improve blood compatibility of AVS materials

Regarding the improvement of blood compatibility of AVS, much can be learned from efforts to make HD membranes with higher blood compatibility,^78–81^ including coating with different modifying molecules. This may cause lower platelet adhesion and activation, lesser clotting with thrombin activation, reduced complement activation, and inflammatory response and enhanced endothelialization.^59,82,83^

Indeed, coating or grafting molecules is a way to improve the blood compatibility of synthetic surfaces and not influencing negatively the mechanical properties of the biomaterials. One of the most used substances to improve hemocompatibility of blood-contacting devices is heparin, which possesses anticoagulant properties by its interaction with anti-thrombin III and heparin-binding protein, blocking thrombin, factor Xa, and other enzymes involved in blood coagulation.^84,85^ Heparin can be grafted to the polymer surface without losing its bioactive properties, improving blood compatibility and cellularization of the graft.^86,87^ Heparin coatings of dialyzers were shown to be effective in reducing blood coagulation in HD.^88–90^ The effect is enhanced using a ligand such as albumin.^90^ The same is valid for heparin-coated ECCs and membrane oxygenators^52,91,92^ and PTFE grafts.^93^ However, the coating is most effective and beneficial if all the inner surfaces of the device are uniformly coated with heparin.^91,92^ Besides limiting the clotting, heparin coating may also reduce the complement activation.^52,91,94^

The thorough coating of heparin on PTFE can be realized by coupling heparin to a PTFE surface coated with dopamine.^95^ Heparin can also be bound to the PTFE surface if it is coated before with a poly(1,8-octanediol-*co*-citrate)^55^ pre-polymer, which is polymerized on the graft surface at 60°C–80°C.^96,97^ In a controlled trial, heparin-bonded grafts demonstrated a non-significant trend to improved patency while it showed a significantly lower early thrombosis rate.^98^ However, these strategies of chemical binding of heparin are more complex and should be designed taking into consideration the ratio of benefit and cost.

Other options to activate inert polymers like PTFE or poly(ethylene terephthalate) (Dacron) are based on the plasma treatment of polymers with ammonia or allyl amine in the gas phase of the reactor,^99^ or N_2_ plasma-immersion-ion-implantation (PIII). The latter technique has the advantage that no chemical cross-linker is used, which would lead to potential toxic side effects.^100,101^ The use of an end-point attachment of heparin (Carmeda™) is another alternative.^93,98,102^ While some^93^ claim superiority of the Carmeda^®^ Bioactive Surface Technology–modified grafts over simple PTFE grafts, other could only find insignificant improvements in the long-term patency but a significantly lower rate of early thrombosis for the first 5 months after implantation.^93,98^ No differences were observed between heparin-bonded (HB-PTFE) grafts and untreated PTFE grafts in a study comprising 483 adult subjects.^75^ The 2-year primary patency rates were ≈20%, primary-assisted patency rates ≈30%, and secondary patency rates ≈37%. Interventions were similar, occurrence of infection (≈11%) and pseudo aneurysm formation (≈5%). Thus, the long-term effect of heparin is still a topic of much debate.^75,97,98^ Are there other synthetic AVS options?

## Synthetic alternatives to PTFE

### Polyurethane

The alternative option to the use of PTFE for AVSs is to explore other materials that can replace it as the dominant graft material. Polyurethane was considered for a time as a replacement material. But while it offers some advantages such as a prompt stop of bleeding at the cannulation site for dialysis, there was no change of the elasticity and mechanical strength for up to 2 years after implantation.^103^ However, it showed an inferior patency rate in comparison with PTFE. Polyurethane also degrades after some time in the human body. Hence, there is a concern about the longevity of the graft beyond 2 years as well as the formation of toxic degradation products such as 2,4-toluene diamine.^104,105^ In vitro data indicate that vascular grafts containing shear stress-conditioned endothelial monolayers maintained the cells better on the surface and were less thrombogenic.^106^ Is there a possibility to use material that allows AVS vessels to mature within a structure that later is degraded and adsorbed?

## Biological and biogenic vascular grafts for AVS

**Figure 5.**
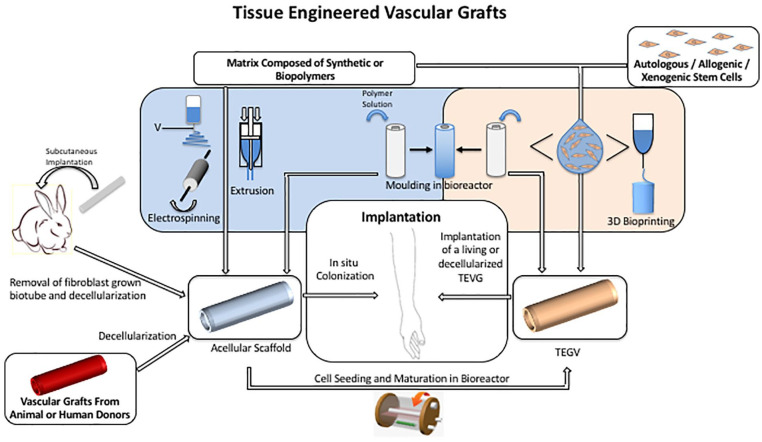
Tissue engineering approaches for developing biological and biogenic vascular grafts for AVS.

Due to the growing number of patients requiring HD treatment and limited VA options, biological or biogenic vascular grafts obtained from decellularized arteries or tissue engineering could help to solve this important clinical problem for HD patients. We describe promising strategies that are currently under investigation and summarized in Figure 5 as follows:

1. *The biodegradable scaffolds* made of synthetic or biopolymers which can be
  (a) Implanted directly “in situ VTE” into the host to promote in vivo remodeling of the scaffold with endogenous cell-recruitment and ECM formation;
  (b) Alternatively, the scaffolds can be seeded with relevant cells in vitro and matured in a bioreactor prior to implantation;
2. *The decellularization* of an allogenic or xenogeneic *artery/vein* which will serve as a scaffold after implantation. These grafts can show late degradation followed by aneurysm formation and immune reaction (intimal hyperplasia);
3. An alternative method to develop vascular grafts is based on the subcutaneous implantation of a compact rod in order to create a foreign body reaction. This reaction leads to the production of a connective tissue covering the implant that can later be used as an autologous tubular vascular graft;
4. Vascular grafts made by *cell assembly (including cell sheets and molding)* are also investigated but are not functional without mixing with the polymer scaffold or a long-term bioreactor maturation to create an ECM. In this way, threads can be obtained from human ECM in order to weave or knit grafts;
5. Bioprinting may provide other ways of manufacturing complex geometry vessels comprising polymers and living autologous or allogenic cells.^107^ Using different cell layers to mimic the native artery or vein;
6. Production of human ECM scaffold or threads: this method requires first the bioreactor’s maturation of human fibroblasts on a rapidly degrading scaffold which is then decellularized in order to obtain a human ECM scaffold which can be used as a so-called “human acellular graft.”

### Tissue-engineered grafts from synthetic or biopolymers

The concept of tissue engineering was first described in the 1980s by the Boston Group (Harvard and MIT) by Langer and Vacanti showing that an engineered living tissue can be created by combining a biodegradable scaffold and cells matured in a bioreactor. While the scaffold degrades the cells rebuild a new ECM, and a new tissue, which are specific to the cells and method used.^108^

The concept has been adapted to vessels and the first vascular tissue engineering (VTE) in-man was performed by Toshi Shinoka who used degradable patches and grafts prepared with cells from patients (during surgery) to operate children with congenital defects such as a large caliber, low pressure, and conduit with good long-term results.^109^

Our group was the first to show that this method could be simplified by omitting the step of cell addition/culture by implanting a biodegradable scaffold directly into the animal as a small caliber arterial replacement (high-pressure system). For this we used a highly porous electro-spun polycaprolactone scaffold made as a vascular structure and could show excellent biocompatibility and mechanical properties over implantation periods up to 2 years in the small and large animals.^110–113^

After implantation of such cell-free scaffold/vessels, the autologous host cells repopulate the scaffold wall and form a confluent endothelial luminal layer, a media with macrophages and myofibroblasts producing a new collagenous ECM while the polymer is degrading. In addition, ingrowing new capillaries provide the blood supply to this “neo artery.”^114,115^

The patency and compliance of such tissue-engineered vessels were better than the classical ePTFE grafts used in clinical practice.^116^

Therefore, we demonstrated a new concept of “in situ VTE” having the advantage to avoid the time-consuming and costly cell-based manufacturing of a new graft and to be shelf-ready and globally applicable to future clinical revascularization procedures.^114,117,118^

Another possibility would be the usage of vascular grafts, grown from human dermal fibroblasts in sacrificial fibrin gel tubes.^119^ First studies on the implantation of such grafts in eight baboons showed 3- and 6-month primary patency of 83% and 60%, respectively, while no graft stenosis was observed. The immune response was only minimal and there was no sign of an aneurysm formation. Although the results are only preliminary, this “off the shelf” graft seems like a promising alternative to PTFE.

### Human, bovine, and other off-the-shelf decellularized vascular grafts

*Decellularized* grafts from human donors were tested in a limited number of patients in early clinical trials with promising results. Such homografts have been used since more than 30 years for cardiovascular replacement materials of infected grafts with good results; however, their number is limited despite the fact that several homograft banks exist throughout the world. More recently and independently, the groups of N. L’Heureux and L. Niklason manufactured in vitro grafts made of human cells.^120,121^ Before implantation, these vascular grafts were decellularized in order to obtain an acellular human matrix scaffold. Such grafts were tested for AVS with similar results to the currently used non-degradable clinical vascular grafts.^121,122^ There is, however, a potential to improve the results obtained with these systems since there is no foreign material and, therefore, they are less vulnerable to infection.^77^ Lawson et al. performed two single-arm phase II trials of their human acellular vascular graft (HAVG) in 60 renal patients requiring HD and demonstrated safety (1 infection in 82 patient-years of follow-up) and efficacy (patency).^121^ The HAVG was mainly composed of human collagens and other natural ECM proteins. Upon implantation, it is anticipated (based on the pre-clinical studies) that the collagen-based matrix comprising the graft will be infiltrated with host cells and remodeled by the host. This process will result in a vascular structure histologically similar to the composition of the native vascular tissue having improved graft longevity and being less susceptible to infection. In the trial, the HAVG was surgically implanted in the forearm or upper arm and the implanted vascular conduit was subsequently used for VA in HD. This HAVG is an alternative to synthetic materials and to autologous grafts in the creation of VA for dialysis (NCT01840956).^123^ Recently, a first ever pivotal, multinational, double-armed, randomized phase III clinical trial has started, aiming to compare the HAVG to the current standard of ePTFE for patients not elective for AVF (NCT02644941).^124^

Other xenogeneic grafts such as bovine carotid artery (BCA) grafts were first reported to be used in clinical applications in the 70s. But, due to their inferior patency, higher cost, and the high occurrence of aneurysms, they fell out of use in favor of other synthetic graft materials like PTFE.^125,126^ A comeback of BCA was able due to new modifications in the collagen crosslinking process and manufacturing of grafts. Marcus et al. reported a comparative study involving 270 patients.^127^ BCA grafts had higher 2-year primary patency (33% vs 14%) and 2-year assisted primary patency rates (57% vs 53%) than PTFE, whereas the 2-year secondary patency rates were similar (BCA vs PTFE = 56% vs 53%). As a PTFE graft cannot be used for 44 ± 16 days after implantation, BCA grafts are generally usable 12 ± 9 days after implantation, which limits another significant failure factor: a TDC. For patients, who need an immediate HD before the PTFE graft is ready, a TDC is employed, which brings its own set of complications, including a risk of invasive infection. The study could show that a TDC-related infection shows up more frequently in PTFE grafts, than in BCA grafts (11 ± 3 vs 5.7 ± 1 per 1000 TDC days).^127^

### Vascular grafts made by cell assembly or bioprinting

The first autologous-biological vascular graft (TEVG), Lifeline™ made by cell assembly used as an AVF for dialysis access was trialed in humans by De la Fuente and Cierpka^128^ (NCT00850252) but, so far, no results are available. These grafts were created using a sheet-based technology, obtained through tissue culture by growing the recipient’s own fibroblast cells taken from a biopsy into a sheet which is then wrapped around a mandrel multiple times and allowed to fuse during incubation. The tube is then seeded with the recipient’s autologous ECs prior to implantation.^129–131^

Another multi-center cohort study investigated the effectiveness of HD access for renal patients using biological and autologous TEVG.^59,132,133^ The results show that their primary patency rate approached established quality objectives for AVF. More recently, the same group followed up with a study using TEVGs built from allogeneic fibroblasts implanted as brachial–axillary AV shunts for three patients requiring HD access.^80^ This case report showed immunological and inflammatory blood markers within normal limits post-implantation, thus opening the field for the use of allogenic human cells for VTE.

### Other options to obtain vascular grafts

Other options are some developments of autologous biotubes^134,135^ a graft, which is grown inside the host by implanting subcutaneously a foreign body precursor in the shape of a compact rod. Through the inflammation process and fibrosis, the ECM is deposited by fibroblasts around the rod and forms a tubular structure. This fibrotic tissue can be used as a vascular graft after explantation of the newly formed tissue and removal of the rod. Tseng et al. demonstrated their potential usage as vascular grafts.^136^ A silicon rod was subcutaneously embedded in New Zealand white rabbits for 1 month after which the biotube was harvested and seeded with adipose-derived stem cells (ADSC). The ADSC differentiated into endothelial and smooth muscle cells through the stimulation of physical blood flow. The biotubes could show 100% patency after 5 months in rabbits and can potentially show a longer resistance to thrombosis and intimal hyperplasia than any of the synthetic grafts. The challenges of this concept are related to the limited wall thickness, since the tissues encapsulate only the surface of the foreign bodies. However, there is already promising research being conducted to optimize this process.^137,138^

Early advances to develop autologous VA conduits progressed at a fast rate, relative to the preceding medical advances. However, the establishment of defined processes to decellularize the vessels to create a truly “off-the-shelf” blood vessel replacement will be absolutely essential to ensure safety, efficacy, and real alternatives for patients.^139^ Tissue engineering technologies have advanced the manufacturing of allogeneic, readily available, bio-mimicking vascular grafts. However, the cost, scale, manufacturing, biocompatibility, thrombogenicity, and durability remain important questions to be answered by this innovative technology. Continued safety and efficacy clinical trials focused on the progression to ensure successful long-term, randomized clinical studies to validate this technology envisioning the needed benefits for millions of patients worldwide.^132^

## Conclusion

Besides optimization of AVF function by various means, the high rate of primary failure and loss of patency in a high percentage of AVF within 2 years after surgery imply the need to use AVS or central venous catheters. There are interesting developments in biomaterials for vascular prosthesis that allow to improve AVS patency. To further improve clinical results, also bioartificial grafts are under investigation and may soon be used in the clinical setting. Combined and interdisciplinary efforts will bring forward new concepts and innovations into the development of high-performance vascular grafts. All these efforts will contribute to enhance the long-term safety and efficacy of AVS as a supplement for AVF in HD access for the benefit of patients as well as for better clinical outcome.
